# Nutritional status affects immune function and exacerbates the severity of pulmonary tuberculosis

**DOI:** 10.3389/fimmu.2024.1407813

**Published:** 2024-07-17

**Authors:** Chunli Lu, Yunyi Xu, Xueya Li, Min Wang, Bei Xie, Yaling Huang, Yan Li, Jiahua Fan

**Affiliations:** ^1^ Department of Clinical Nutrition, Guangzhou Chest Hospital, Guangzhou, China; ^2^ Department of Clinical Laboratory, Guangzhou Chest Hospital, Guangzhou, China; ^3^ Department of Tuberculosis, Guangzhou Chest Hospital, Guangzhou, China; ^4^ Department of Institute of Tuberculosis, Guangzhou Chest Hospital, Guangzhou, China; ^5^ State Key Laboratory of Respiratory Disease, Department of Clinical Nutrition, Guangzhou Chest Hospital, Institute of Tuberculosis, Guangzhou Medical University, Guangzhou, China; ^6^ Guangzhou Key Laboratory of Tuberculosis Research, Department of Clinical Nutrition, Guangzhou Chest Hospital, Institute of Tuberculosis, Guangzhou Medical University, Guangzhou, China

**Keywords:** nutritional status, immune function, pulmonary tuberculosis (PTB), BMI, mediation

## Abstract

**Aim:**

To comprehensively evaluate the association and impact of nutritional status and immune function on the severity of pulmonary tuberculosis (PTB).

**Methods:**

This descriptive cross-sectional study involved 952 participants who were diagnosed with active PTB. Severe PTB involves three or more lung field infections based on chest radiography. Nutritional status was evaluated using various indicators, including body mass index (BMI), the nutritional risk screening score (NRS-2002), total protein (TP), prealbumin (PA), transferrin (TRF), and serum albumin (ALB) levels and the prognostic nutritional index (PNI). Immune dysfunction was defined as a CD4^+^ count <500 cells/µl or a CD4^+^/CD8^+^ ratio <1. Neutrophil-to-lymphocyte ratio (NLR) and platelet-to-lymphocyte ratio (PLR) were also calculated. Multivariate logistic and generalized linear regression were used to assess the associations between nutritional status, immune function, the severity of PTB, and the number of infected lung fields, adjusting for age, sex, and diabetes. Mediation analysis was conducted to evaluate the extent to which immune function mediated the impact of nutritional status on the severity of PTB. Sensitivity analysis was performed to enhance the robustness of the results.

**Results:**

Compared to those in the general PTB group, patients in the severe PTB group tended to be older men with diabetes. Higher nutritional risk, higher proportion of immune dysfunction and lower lymphocyte counts were observed in the severe group. BMI and the PNI were found to be protective factors, while PLR was identified as a risk factor for disease severity. Immune dysfunction and the PLR are mediators of the relationship between nutritional status and PTB severity. When BMI, the PNI, and the PLR were combined with traditional clinical indicators, these parameters showed promising diagnostic value, and the AUC reached 0.701 (95% CI: 0.668–0.734).

**Conclusion:**

The findings suggest that nutritional status is significantly associated with the severity of PTB, and immune function mediates the effects of nutritional status on the severity of PTB. Maintaining adequate BMI, PNI levels, and immune function or reducing PLR levels helps reduce the risk of severe PTB.

## Introduction

Tuberculosis is an ancient infectious disease in human history that poses a serious global public health problem. The Global Tuberculosis Report released by the World Health Organization in 2023 revealed that in 2022 (1), there were 10.6 million new cases of tuberculosis worldwide, with an incidence rate of 133 per 100,000 people. In 2022, tuberculosis ranked as the second leading infectious cause of death globally, following COVID-19, with 1.3 million deaths attributed to tuberculosis. Pulmonary tuberculosis (PTB) accounts for the majority of tuberculosis cases, and a small percentage of patients with PTB may require intensive care unit (ICU) admission for complex treatment due to complications such as multiorgan failure, respiratory failure, septic shock, and acute kidney injury (2). Compared to most patients with ordinary PTB, critically ill patients with PTB have a significantly higher mortality rate (3, 4). Enhancing the prognosis of critically ill patients with PTB is essential for reducing TB-related mortality.

Current research indicates that immune and nutritional status are important factors influencing the clinical prognosis of tuberculosis patients (5, 6). Various studies have indicated that various types of inflammatory cytokines of both the innate and adaptive immune systems coordinate the immune response of an individual to *Mycobacterium tuberculosis* infection, and nutritional intervention is associated with a substantial reduction in the incidence of tuberculosis in households (7). Parameters such as the neutrophil-to-lymphocyte ratio (NLR), platelet-to-lymphocyte ratio (PLR), and T lymphocyte subsets serve as key indicators of immune status, reflecting the body’s inflammatory response and immune equilibrium. In addition to conventional indicators such as body mass index (BMI), total protein (TP), prealbumin (PA), and transferrin (TRF) for assessing the nutritional status of the body, serum albumin levels, the nutritional risk screening score (NRS-2002), and the prognostic nutritional index (PNI) are pivotal markers for evaluating nutritional and immune status. While existing research has delved into the link between nutritional status, immune function, and tuberculosis severity (5, 6), large sample size cohort studies simultaneously exploring the joint impact of nutrition and immunity on tuberculosis severity remain limited. A comprehensive investigation of how nutrition and immunity influence the severity of PTB is still lacking.

Therefore, this study aimed 1) to examine the relationship between nutrition, immunity, and the severity of PTB and 2) to determine the nutritional and immune function indexes related to the early identification of PTB severity and their discrimination ability.

## Methods

### Study cohort

This study included 952 inpatients who were diagnosed with active PTB at Guangzhou Chest Hospital between January and December 2023. The inclusion criteria for patients were as follows: 1) met the diagnostic criteria for PTB and 2) met the diagnostic criteria for active PTB. The exclusion criteria were as follows: 1) patients with malignant tumors and 2) patients with systemic immune diseases such as lupus erythematosus or HIV-positive individuals. General patient information, including sex, age, BMI, smoking status, basic disease status, disease course, treatment history, initial pulmonary imaging characteristics, complete blood count, lymphocyte subsets, biochemical test results, etc., was collected. This study was approved by the Medical Ethics Committee of Guangzhou Chest Hospital (2022–90), and written informed consents were obtained from each individual.

### Definition of severe pulmonary tuberculosis

The degree of pulmonary involvement, hemoglobin level, age and comorbidities of PTB patients are the key factors affecting the severity of PTB (4). Moreover, since the serum ALB concentration decreases significantly with increasing percentage of the lung field infectious area (8), the number of infected lung fields ≥3 was the only independent predictor of shorter survival in patients with tuberculous lung destruction. Therefore, this study defined severe PTB as active PTB with the number of infected lung fields ≥3 lung fields on chest imaging. All patients were divided into the severe PTB group (number of infected lung fields ≥3) (n=519) and a non-severe PTB group (number of infected lung fields <3) (n=433) based on their radiographic findings.

### Assessment of the nutritional and immune status of patients

The subjects in this study underwent nutritional risk screening using the NRS-2002 (9) during hospitalization. The screening was conducted according to the standards of “WS/T 427–2013 Clinical Nutrition Risk Screening”, with scoring based on three components: nutritional status score, disease severity score, and age score. The final score is the sum of these three scores. For the nutritional status score and disease severity score, the highest score in each category was used as the final score for that category. Individuals with an NRS-2002 score of ≥3 are considered to have nutritional risk (10), while those with a score of <3 are considered to have no nutritional risk. In addition, body mass index (BMI), total protein (TP), prealbumin (PA), transferrin (TRF), and serum albumin (ALB) levels were collected.

For immune status, those with a CD4^+^ count <500 cells/µl or a CD4^+^/CD8^+^ ratio <1 were regarded as having immune dysfunction, while individuals with a CD4^+^ count ≥500 cells/µl and a CD4^+^/CD8^+^ ratio ≥1 were considered to have normal immune function. The neutrophil-to-lymphocyte ratio (NLR) and platelet-to-lymphocyte ratio (PLR) were calculated, and prognostic nutritional index (PNI) was calculated as follows: PNI = serum albumin (g/L) + 5 × peripheral blood lymphocyte count (×10^9^/L).

### Immune cell assay

The subjects fasted overnight, and 3 mL of fasting antecubital venous blood was collected in EDTA-K anticoagulation tubes for the CD4^+^/CD8^+^/CD3^+^ lymphocyte subpopulation technique to determine the immune cell content. Utilizing a proprietary original single-cell-specific solid-phase capture technology, human blood samples were targeted for immune cell capture using monoclonal antibodies coated on immunocytochemically stained slides. Immune cells such as CD3^+^, CD4^+^, and CD8^+^ cells were captured through antigen-antibody binding reactions. Subsequently, with the combination of chemical staining and computer image analysis techniques, visualization analysis of immune cells and precise absolute counting were achieved to comprehensively evaluate the body’s cellular immune function. The BEION M4-BF biological microscope used in this study was obtained from Shanghai Beion Medical Technology Co., Ltd., China.

### Statistical analysis

Statistical analyses were conducted using R software, version 4.0.3, unless otherwise specified. Non-normally distributed continuous data were expressed as median (M) and interquartile range (P25, P75), and analyzed using the Mann-Whitney U test. Categorical data were presented as frequencies (percentages) and analyzed using the chi-squared test. Multifactorial logistic regression and generalized linear models were used to analyze the risk factors associated with severe PTB and an increase in the number of infected lung fields in tuberculosis patients. The *R*-square of the model is used to indicate the percentage of variation in the severity of PTB or the number of pulmonary fields infected with tuberculosis explained jointly by nutritional or immune function indicators (11, 12). Receiver operating characteristic (ROC) curves were used to evaluate the diagnostic performance of single indicators and combined tests. A significance level of *P*<0.05 was considered to indicate statistical significance.

### Random forest model

All indicators of nutritional or immune status were used separately or together to construct a random forest model with the R package *randomForest* V4.7–1.1 with the default settings to explore important indicators contributing to the classification of subjects with or without severe tuberculosis. Feature importance was evaluated by SHapley Additive exPlanations with the R package *DALEX* V2.4.3 and visualized with the R package *shapviz* V0.9.0. The subjects were randomly split at a 3:1 ratio for model training and verification. For clarity, only the top 10 indicators are shown in the figure.

### Mediation analysis

To evaluate whether immune status mediated the impact of nutritional status on the severity of tuberculosis or the number of pulmonary fields infected with tuberculosis, we applied causal mediation analysis using the mediate function from the R package mediation. We estimated the total effect, direct effect and indirect effect of immune status or nutritional status on the severity of tuberculosis or the number of pulmonary fields infected with tuberculosis from these models. The results were confirmed by 100 bootstrap replicates of the simulation.

## Results

### Baseline characteristics based on the severity of tuberculosis

**Table 1** shows the baseline characteristics based on the severity of tuberculosis. Overall, the cohort population aged between 34 and 64 years, with a median age of 52 years. There were 67.8% of the patients were male, with 519 individuals rated to have severe PTB, accounting for approximately 54.52% of the total population. Compared to individuals in the general PTB group, those in the severe PTB group were more likely to be older men with diabetes, regardless of their historical smoking status, with a higher proportion of mear-positive sputum, a longer course of the disease, and a greater bacillary load. In terms of indicators related to nutritional status, the severe PTB group had a higher proportion of patients at nutritional risk, higher NRS-2002 scores, and lower BMI, PNI, ALB, PA, and TRF. Regarding indicators related to immune status, a greater proportion of individuals in the severe PTB group had immune dysfunction, especially a significantly higher percentage of individuals with a CD4^+^ T-cell count <500 cells/µl than did individuals in the general PTB group. Additionally, patients in the severe PTB group had lower CD4^+^, CD8^+^, CD3^+^, and lymphocyte counts and higher NLRs and PLRs. These results suggest that compared to those in the general PTB group, patients in the severe PTB group had poorer nutritional status, weaker immune function, and more severe inflammatory conditions.

**Table 1:** Characteristics of participants by tuberculosis severity (n = 952).

Characteristic	Total Population	Number of infected lung fields < 3	Number of infected lung fields ≥ 3	*P* value
	(*n*=952)	(*n*=433)	(*n*=519)	
Male, n (%)	645 (67.8%)	269 (62.1%)	376 ( 72.4%)	0.001
Age (years)	52.00 [34.00, 64.00]	48.00 [32.00, 61.00]	54.00 [35.00, 65.00]	<0.001
Current smoking, n (%)	196 (20.6%)	94 (21.7%)	102 ( 19.7%)	0.469
With diabetes, n (%)	317 (33.3%)	128 (29.6%)	189 ( 36.4%)	0.027
Nutritional status				
Nutritional risk, n (%)	440 (46.2%)	165 (38.1%)	275 ( 53.0%)	<0.001
NRS score (2002)	2.00 [1.00, 3.00]	2.00 [1.00, 3.00]	3.00 [1.00, 4.00]	<0.001
BMI ^a^	20.15 [18.70, 22.50]	20.80 [18.80, 22.90]	19.70 [18.60, 21.60]	<0.001
PNI (g/l)	43.70 [36.19, 49.30]	46.70 [40.05, 50.60]	41.10 [34.10, 46.83]	<0.001
TP (g/l) ^a^	70.23 [64.72, 75.36]	71.08 [65.38, 75.46]	69.52 [64.47, 75.16]	0.092
ALB (g/l) ^a^	36.70 [30.60, 42.00]	39.00 [33.40, 42.90]	34.60 [29.15, 39.85]	<0.001
PA (g/l) ^a^	204.61 [150.17, 252.05]	217.10 [164.60, 268.83]	192.80 [141.96, 240.48]	<0.001
TRF (g/l) ^a^	210.25 [164.48, 260.80]	220.39 [180.75, 268.76]	198.34 [155.83, 251.87]	<0.001
Immune function				
Immunocom promise, n (%)	526 (55.3%)	205 (47.3%)	321 ( 61.8%)	<0.001
CD4^+^<500, n (%)	513 (53.9%)	198 (45.7%)	315 ( 60.7%)	<0.001
CD4^+^/CD8^+^<1, n (%)	86 ( 9.0%)	32 ( 7.4%)	54 ( 10.4%)	0.113
CD4^+^ (cells/ul)	476.00 [328.00, 664.00]	524.00 [372.00, 744.00]	436.00 [296.00, 604.00]	<0.001
CD8^+^ (cells/ul)	276.00 [181.50, 409.00]	304.00 [204.00, 444.00]	256.00 [168.00, 379.50]	<0.001
CD3^+^ (cells/ul)	796.00 [555.00, 1104.00]	876.00 [612.00, 1220.00]	744.00 [514.00, 1014.00]	<0.001
CD4^+^/CD8^+^	1.69 [1.30, 2.21]	1.69 [1.33, 2.16]	1.68 [1.27, 2.28]	0.822
LYM (10^9^/L)	1.24 [0.89, 1.64]	1.32 [0.99, 1.74]	1.16 [0.79, 1.56]	<0.001
NLR	3.78 [2.44, 6.39]	3.34 [2.23, 5.22]	4.28 [2.66, 7.58]	<0.001
PLR	218.56 [151.65, 334.88]	195.28 [135.46, 275.00]	244.23 [168.29, 390.80]	<0.001
Tuberculosis severity				
Number of pulmonary fields infected with tuberculosis	3.00 [2.00, 6.00]	2.00 [1.00, 2.00]	6.00 [6.00, 6.00]	<0.001
Smear-positive sputum (%)	378 ( 39.7%)	123 ( 28.4%)	255 ( 49.1%)	<0.001
Bacillary load (%)				<0.001
–	574 ( 60.3%)	310 (71.6%)	264 ( 50.9%)	
±	63 ( 6.6%)	24 ( 5.5%)	39 ( 7.5%)	
1^+^	87 ( 9.1%)	33 ( 7.6%)	54 ( 10.4%)	
2^+^	83 ( 8.7%)	22 ( 5.1%)	61 ( 11.8%)	
3^+^	75 ( 7.9%)	25 ( 5.8%)	50 ( 9.6%)	
4^+^	70 ( 7.4%)	19 ( 4.4%)	51 ( 9.8%)	
PTB combined with extrapulmonary tuberculosis (%)	480 ( 50.4%)	210 ( 48.5%)	270 ( 52.0%)	0.298
Course of disease (month)	6.00 [2.00, 12.00]	6.00 [2.00, 12.00]	7.00 [3.00, 24.00]	<0.001
Drug resistant cases (%)	397 ( 41.7%)	175 ( 40.4%)	222 ( 42.8%)	0.469

a These variables had missing values. Number of participants with missing value were as follow: BMI (n = 38), TP (n = 4), ALB (n = 4), PA (n = 176), and TRF (n = 176). The above missing values were interpolated according to the random forest multiple interpolation method.

### Associations between nutritional or immune status and tuberculosis severity

**Figure 1:**
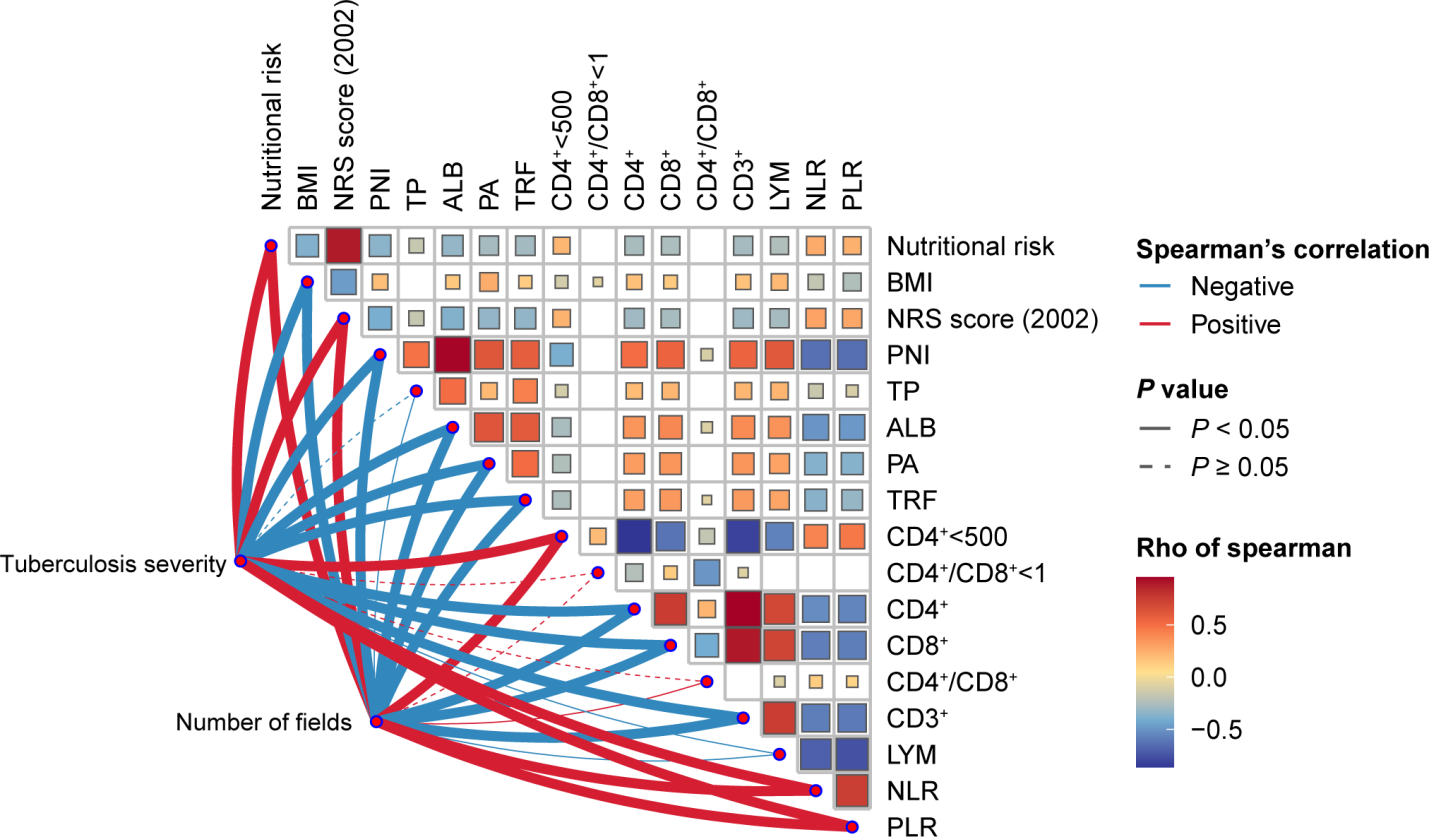
Heatmap of the Spearman’s correlation coefficients between nutritional status and immune status indices and the severity of tuberculosis. Red and blue indicate positive and negative associations, respectively.

Initially, through Spearman correlation analysis (**Figure 1**), we found multiple significant correlations among nutritional and immune status indicators. For instance, nutritional risk was significantly correlated with immune dysfunction (CD4^+^ < 500 cells/µl), NLR and PLR, but significantly negatively correlated with CD4+, CD8+, CD3+, and LYM counts. Additionally, immune dysfunction (CD4^+^ < 500 cells/µl) exhibited negative correlations with BMI, PNI, TP, ALB, PA, and TRF. Furthermore, apart from CD4^+^/CD8^+^<1, CD4^+^/CD8^+^, TP, and LYM, for which no significant differences were observed, the remaining nutritional and immune status indicators showed significant associations with the severity of PTB and the number of infected lung fields.

Next, we included indicators that showed significant correlations with the severity of PTB and the number of infected lung fields in a multifactorial logistic regression analysis to explore which indicators related to nutritional and immune status were associated with the severity of PTB. **Table 2** shows that BMI and the PNI are independent protective factors for the occurrence of severe PTB in patients, while the PLR is an independent risk factor. According to further generalized linear model analysis with the number of infected lung fields as the dependent variable, we found that BMI and PNI were also found to be independent protective factors for delaying the progression of the number of infected lung lobes, while the PLR was also a risk factor. The associations of BMI, the PNI, and the PLR with the severity of PTB symptoms and the number of infected lung fields remained statistically significant after adjusting for age, sex, BMI, diabetes and course of disease.

**Table 2:** Association between nutritional or immune status and tuberculosis severity.

Variables		Unadjusted				Adjusting for sex, age, diabetes and course of disease			
		*β*	Wald	OR (95% CI)	P value	*β*	Wald	OR (95% CI)	P value
Nutritional status									
Tuberculosis severity (the number of infected lung fields ≥ 3)	BMI	-0.080	9.339	0.923(0.877~0.971)	0.002	-0.099	12.576	0.905(0.856~0.956)	<0.001
	NRS	0.078	1.471	1.081(0.953~1.227)	0.225	0.049	0.564	1.051(0.923~1.196)	0.453
	PNI	-0.070	7.867	0.932(0.887~0.979)	0.005	-0.062	5.728	0.940(0.893~0.988)	0.017
	ALB	0.028	0.879	1.028(0.971~1.090)	0.349	0.039	1.688	1.039(0.981~1.102)	0.194
	PA	0.000	0.124	1.000(0.998~1.003)	0.724	0.000	0.001	1.000(0.998~1.002)	0.975
	TRF	0.000	0.004	1.000(0.998~1.002)	0.951	0.001	0.312	1.001(0.998~1.003)	0.577
Number of pulmonary fields infected with tuberculosis	BMI	-0.079	9.672	0.924(0.879~0.971)	0.002	-0.110	17.912	0.895(0.851~0.942)	<0.001
	NRS	0.104	2.712	1.110(0.980~1.257)	0.100	0.059	0.875	1.061(0.937~1.202)	0.350
	PNI	-0.014	4.504	0.986(0.973~0.999)	0.034	-0.015	4.927	0.986(0.973~0.998)	0.027
	ALB	-0.041	8.094	0.960(0.934~0.987)	0.005	-0.022	2.199	0.979(0.951~1.007)	0.138
	PA	0.001	0.379	1.001(0.998~1.003)	0.538	0.000	0.012	1.000(0.998~1.002)	0.912
	TRF	-0.002	1.658	0.998(0.996~1.001)	0.198	-0.001	0.374	0.999(0.997~1.002)	0.541
Immune status									
Tuberculosis severity (the number of infected lung fields ≥ 3)	CD4^+^	0.004	1.147	1.004(0.999~1.014)	0.284	0.004	0.788	1.004(0.999~1.013)	0.375
	CD8^+^	0.004	0.999	1.004(1.000~1.013)	0.318	0.004	0.841	1.004(1.000~1.013)	0.359
	CD3^+^	-0.004	1.233	0.996(0.987~1.000)	0.267	-0.004	0.898	0.996(0.987~1.001)	0.343
	NLR	0.034	2.696	1.034(0.995~1.078)	0.101	0.022	1.200	1.022(0.984~1.066)	0.273
	PLR	0.001	5.403	1.001(1.000~1.002)	0.020	0.001	6.014	1.001(1.000~1.003)	0.014
Number of pulmonary fields infected with tuberculosis	CD4^+^	0.002	2.727	1.002(1.000~1.004)	0.099	0.001	0.727	1.001(0.999~1.003)	0.394
	CD8^+^	0.001	1.995	1.001(1.000~1.002)	0.158	0.001	1.327	1.001(0.999~1.002)	0.250
	CD3^+^	-0.002	4.091	0.998(0.997~1.000)	0.043	-0.001	1.536	0.999(0.997~1.001)	0.216
	NLR	0.040	5.822	1.040(1.007~1.074)	0.016	0.026	2.519	1.026(0.994~1.059)	0.113
	PLR	0.001	5.652	1.001(1.000~1.002)	0.018	0.001	6.926	1.001(1.000~1.002)	0.009

OR, Odds ratio.

CI, Confidence interval.

**Figure 2:**
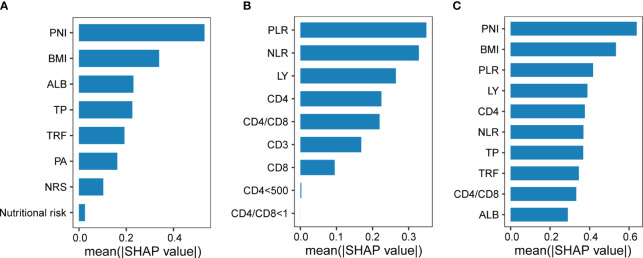
The efficacy of nutritional and immune status in diagnosing the severity of pulmonary tuberculosis. Indicators of nutritional status **(A)** or immune status **(B)** contributing to the classification of subjects with or without severe PTB, respectively. **(C)** Indicators of nutritional status or immune status contributing to the classification of subjects with or without severe tuberculosis.

Moreover, we found that a machine learning algorithm integrating nutritional and immune indicators could differentiate severe PTB patients from general PTB patients. Whether incorporating nutritional and immune indicators separately or together into the model, the information features contributing the most to this classifier included BMI, PNI, and PLR (**Figures 2A-C**). Therefore, by combining multiple linear models and random forest machine learning algorithms, we believe that BMI, the PNI, and the PLR are important indicators related to the severity of PTB in terms of nutritional and immune status.

### Nutritional status exacerbates tuberculosis severity by affecting immune function

**Figure 3:**
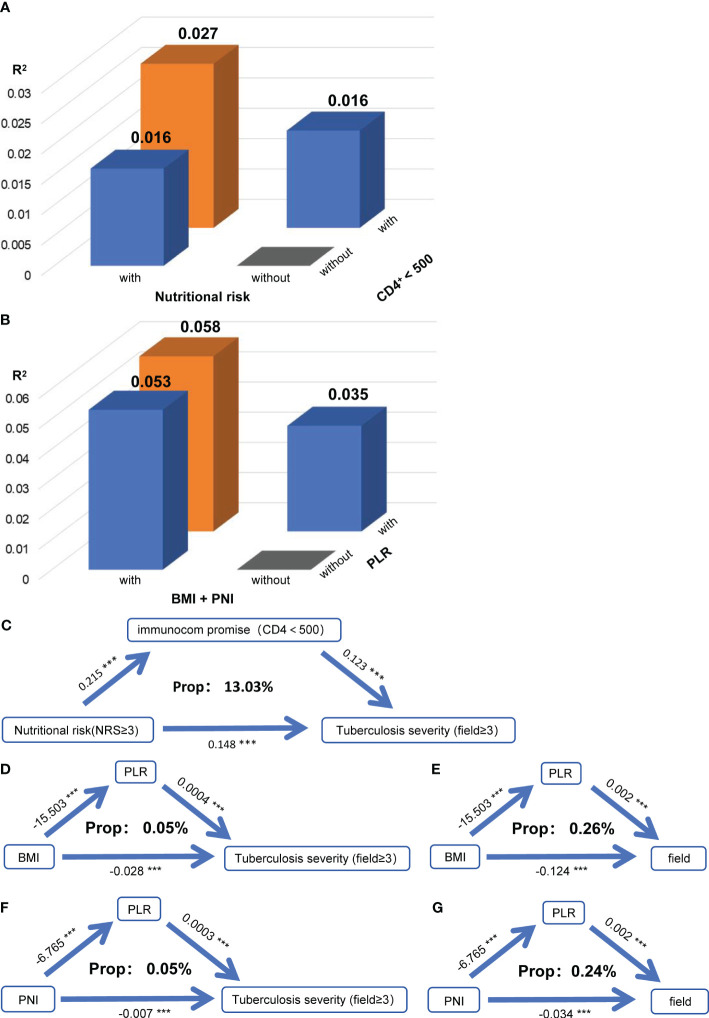
Nutritional status exacerbates tuberculosis severity by affecting immune function. **(A)** When the severity of PTB was used as the dependent variable in the model, the R square of the model for nutritional risk and immune dysfunction (CD4^+^<500) were used as independent variables separately or simultaneously. **(B)** When the severity of PTB was used as the dependent variable in the model, the R square of the model for the nutritional index (BMI + PNI) and immune index (PLR) were used as independent variables separately or simultaneously. **(C)** Mediation linkage between nutritional risk and immunocom promise for tuberculosis. Relationships between BMI and the PLR and between PTB severity **(D)** and the number of infected lung fields **(E)**. Relationships between the PNI and PLR and between PTB severity **(F)** and the number of infected lung fields **(G)**. Indirect effects, *P* mediation, and the mediatory effect of each metabolite are denoted. **P<0.01

Subsequently, we found that when nutritional risk and immune dysfunction, as well as indicators of nutritional and immune status, were simultaneously included in the model, variation in the severity of PTB was explained to a higher degree (R^2^ with nutritional risk + immune dysfunction: 0.027, and R^2^ with BMI + PNI + PLR: 0.058; **Figures 3A, B**). This result suggested that nutritional and immune status may interact and influence each other’s impact on the severity of PTB. However, we found no interaction between nutritional risk and immune dysfunction or between BMI or the PNI and the PLR (**Supplementary Table S1**).

To further explore how nutritional and immune status jointly influence the severity of PTB, we conducted a mediation analysis between nutritional risk and immune dysfunction, and between BMI and PNI with PLR. We discovered that immune dysfunction mediated the impact of nutritional risk on the severity of PTB, with a mediation proportion of 13.03% (**Figure 3C**). Additionally, PLR mediated the effects of BMI (**Figures 3D, E**) and PNI (**Figures 3F, G**) on the severity of PTB and the number of infected lung fields. While no interaction effects were present, the mediation effects indicated that nutritional status may influence the severity of PTB and the number of infected lung fields by modulating immune function.

### The efficacy of nutritional and immune status in diagnosing the severity of pulmonary tuberculosis

**Figure 4:**
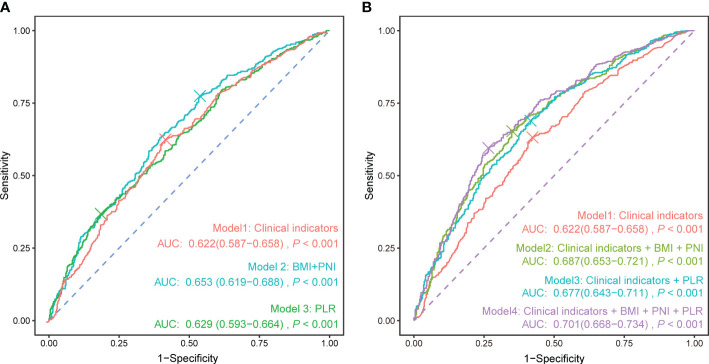
ROC curve of each indicator for the diagnosis of severe pulmonary tuberculosis. **(A)** Clinical indicators, nutritional indicators, and immune status indicators were used separately to calculate the ROC curve. **(B)** Based on the clinical indicators, nutritional and immune status indicators were sequentially added to calculate the ROC curve. Clinical indicators: sex + age + diabetes + current smoking + retreatment.

To investigate whether nutritional and immune status can improve the ability to differentiate between severe PTB patients and general PTB patients based on commonly used clinical indicators, we analyzed the diagnostic performance of BMI, the PNI, the PLR, and common clinical indicators for severe PTB using ROC curves (**Figure 4**). The sensitivity and specificity of the model using common clinical indicators alone (**Figure 4A**) were 63.2% and 57.7%, respectively, with an area under the curve (AUC) value of 0.622 (95% CI: 0.587–0.658). The AUC for diagnosing severe PTB using BMI and the PNI was 0.653 (95% CI: 0.619–0.688), while the sensitivity and specificity of diagnosing severe PTB using the PLR were 36.8% and 81.5%, respectively, with an AUC of 0.629 (95% CI: 0.593–0.664). The AUC values of these two models were higher than those of the model using common clinical indicators alone, indicating that the selected nutritional and immune status indicators had a better ability to differentiate between severe PTB patients and general PTB patients than did the common clinical indicators.

Furthermore, adding nutritional and immune status indicators separately on the basis of common clinical indicators could enhance the ability to differentiate patients to different extents (**Figure 4B**), with AUC values reaching 0.687 (95% CI: 0.653–0.721) and 0.677 (95% CI: 0.643–0.711), respectively. When BMI, the PNI, the PLR, and common clinical indicators were used together to differentiate patients, the AUC reached 0.701 (95% CI: 0.668–0.734), showing certain preliminary diagnostic value for severe pulmonary TB in clinical practice.

### Sensitivity analysis

To avoid errors caused by missing clinical data, we repeated all analyses after excluding patients with missing clinical data. Subsequently, the association between BMI, the PNI, and the PLR and the severity of PTB remained unchanged (**Supplementary Table S2**). Furthermore, immune dysfunction also mediated the impact of nutritional risk on the severity of PTB (**Supplementary Figure S1A**), while the PLR similarly mediated the effects of BMI and PNI on the severity of PTB and the number of infected lung fields (**Supplementary Figure S1B-E**).

There is no definitive criterion for the severity of PTB, and whether PTB is complicated by cavities is also a clinically significant indicator of tuberculosis severity (13). Therefore, we regrouped the population based on the presence or absence of cavities and divided them into a cavity group (n = 473) and the non-cavity group (n = 479) (**Supplementary Table S3**) to explore whether the use of different indicators for assessing the severity of PTB affected the conclusions. BMI and CD8^+^ status were protective factors against the severity of PTB (**Supplementary Table S4**), and CD8^+^ status mediated the impact of BMI on the severity of PTB (**Supplementary Figure S2A**). In addition, bacillary load is also an important indicator of the tuberculosis severity. Therefore, we also regrouped the population based on sputum culture results and divided them into smear-positive sputum group (n = 378) and smear-negative sputum group (n = 574) (**Supplementary Table S5**) to further explore whether the use of indicators other than X-ray images for assessing the severity of PTB affected the conclusions. ALB and CD3^+^ status were protective factors against the severity of PTB (**Supplementary Table S6**). Immunocom promise mediated the impact of nutritional risk on the severity of PTB (**Supplementary Figure S3A**), and CD8^+^, CD3^+^, NLR or PLR status mediated the impact of ALB or NRS on the severity of PTB, separately (**Supplementary Figure S3B-H**). These suggest that although the way in which immune status mediates the impact of nutritional status on the severity of PTB may change with different indicators of tuberculosis severity, nutritional status can still affect the severity of PTB by regulating immune function.

Since being a new treatment patient may affect the severity of PTB, we further stratified the analysis based on whether the patients were newly treated and sex. We found that in both newly treated patients and retreated patients, BMI and the PLR were significantly correlated with the severity of PTB (**Supplementary Table S7**), and the PLR similarly mediated the effects of BMI on the severity of PTB and the number of infected lung fields (**Supplementary Figure S4A-D**).

The results of the sensitivity analysis above all suggest that our conclusions are robust.

## Discussion

In this study, cohort of tuberculosis patients, we investigated the associations between nutritional and immune status and the severity of PTB and further explored how nutritional and immune status jointly influence the severity of PTB. Our research confirmed previous findings indicating the relevance of nutritional and immune status to the severity of PTB. Additionally, our study revealed that BMI and the PNI act as protective factors for the severity of PTB, while the PLR serves as a risk factor. Furthermore, we found that immune dysfunction mediated the impact of nutritional risk on the severity of PTB, and the PLR mediated the effects of BMI and PNI on the severity of PTB and the number of infected lung fields.

Overall, our study is consistent with existing research results. In our correlation analysis involving nutritional status, immune function, and the severity of PTB, we observed numerous significant relationships among indicators of nutrition and immune status. Specifically, there was a positive correlation between nutritional risk and immune dysfunction, along with positive associations with the NLR and PLR, and negative associations with CD4^+^, CD8^+^, CD3^+^, and LYM counts. Previous research findings suggest that malnutrition can impact both innate and adaptive immunity (14) and that patients with PTB who use immunosuppressants are at heightened nutritional risk (15), all of which align with our conclusions. Moreover, the correlations among BMI, the PLR, and the PNI are consistent with previous research, indicating that a lower PNI is associated with a lower BMI and a higher PLR (16).

Additionally, we found that key nutritional indicators, such as the PNI and BMI, act as protective factors against the severity of PTB, whereas the immune function indicator PLR functions as an independent risk factor for disease severity. These results are in accordance with current research suggesting that nutrition and immune status play pivotal roles in influencing the clinical outcomes of tuberculosis patients (5). This finding suggested that nutrition and immune status could partly influence the severity and extent of lung infection in tuberculosis patients. Moreover, previous literature indicates that the PNI has been extensively investigated in chronic conditions such as vascular and kidney diseases, where systemic inflammation plays a role in the progression of the disease and unfavorable outcomes (17–19). The results of our research suggest that the PNI reflects the nutritional and immune states of patients and potentially serves as an indicator of tuberculosis severity by reflecting systemic inflammation and overall physiological status. Additionally, we observed a significant positive correlation between the presence of < 500 CD4+ cells/µl and the severity of PTB. In contrast, the association between CD4^+^/CD8^+^ < 1 and tuberculosis severity was found to be nonsignificant. This implies that when assessing immune function indicators in tuberculosis patients, a CD4^+^ T-cell count of less than 500 cells/µl may be more practically valuable than a CD4^+^/CD8^+^ T-cell ratio of less than 1.

Furthermore, our research highlights that BMI is a robust indicator of nutritional status, demonstrating a significant negative association with the severity of tuberculosis, regardless of the extent of lung involvement, the presence of lung cavities, or the status of being a new tuberculosis patient as a parameter for assessing disease severity. The literature supports the notion that body mass index (BMI) is a reliable tool for evaluating an individual’s nutritional status. In different TB burden settings, there is a negative logarithmic relationship between BMI and the incidence rate of TB, with a 14% decrease in TB incidence for each unit increase in BMI (20). Moreover, there is a connection between BMI and inflammatory cytokines in patients with tuberculosis (21). Circulating levels of proinflammatory cytokines are positively correlated with BMI (between 25 and 29.9 kg/m^2^) and negatively correlated with the levels of anti-inflammatory cytokines in individuals with lower BMIs (22, 23). These results indicate that BMI acts as a protective mechanism against the progression of tuberculosis infection to disease by altering an individual’s cytokine environment. Our study suggested that BMI is significantly negatively correlated with the severity of PTB, regardless of whether the extent of pulmonary involvement in tuberculosis infection or relapse status is used as the criterion for severity assessment. Additionally, the nutritional-immune indicator PLR mediates the impact of BMI on the severity of PTB. Furthermore, several studies have indicated that supplementation of TB patients with micronutrients/macronutrients not only improves body weight and BMI but also affects T-cell function, sputum conversion, relapse, physical activity, and mortality (24–31). These findings collectively support the notion that BMI may serve as a crucial nutritional indicator for the early diagnosis of the severity of PTB.

Although the biological mechanism by which BMI regulates immune status and subsequently influences the severity of tuberculosis remains a mystery, the literature suggests that the function of various antigen-presenting cell types, such as B lymphocytes, macrophages, dendritic cells (DCs), and Kupffer cells, decreases during malnutrition (32), which may lead to a decrease in the body’s resistance to *Mycobacterium tuberculosis*, thereby increasing the severity of the disease. Additionally, there is evidence indicating a connection between BMI and inflammatory cytokines in patients with tuberculosis. Individuals with a low BMI have decreased levels of proinflammatory cytokines (IFN-γ, TNF-α, IL-22, IL-1α, IL-1β, and IL-6) but increased levels of regulatory cytokines (IL-10, TGF-β, IL-5, and IL-13) (21), suggesting a potential biological mechanism by which BMI regulates the severity of TB through immune modulation.

Our study has some limitations worth mentioning. Our study is a cross-sectional study, and causality cannot be established. Follow-up studies will be conducted on newly diagnosed patients to explore their long-term prognosis. Despite these limitations, our study has several strengths, including the large number of subjects and significant control of confounding factors. Additionally, we utilized sensitivity analysis to address issues such as missing data, unclear criteria for assessing the severity of PTB, and retreatment, enhancing the robustness of our study conclusions. Furthermore, for the first time, we employed a mediation analysis to investigate the relationships among nutritional status, immune function, and the severity of PTB, suggesting that immune dysfunction may mediate the impact of nutritional risk on the severity of PTB.

In conclusion, our study provides evidence that nutritional status is significantly associated with the severity of PTB, and immune function mediates the effects of nutritional status on the severity of PTB. Maintaining adequate BMI, PNI levels, and immune function or reducing PLR levels helps reduce the risk of severe PTB. This result suggests the importance of paying attention to nutritional status in the diagnosis and treatment of patients with PTB and that proactive nutritional therapy may help improve the prognosis and severity of tuberculosis infection in patients. At the same time, nutritional and immune status indicators are expected to become early diagnostic markers for the severity of tuberculosis.

## Data availability statement

The original contributions presented in the study are included in the article/**Supplementary Material**. Further inquiries can be directed to the corresponding authors.

## Ethics statement

The studies involving humans were approved by the Medical Ethics Committee of Guangzhou Chest Hospital (2022-90). The studies were conducted in accordance with the local legislation and institutional requirements. Written informed consent for participation in this study was provided by the participants’ legal guardians/next of kin.

## Author contributions

CL: Conceptualization, Data curation, Formal analysis, Investigation, Methodology, Visualization, Writing – original draft, Writing – review & editing. YX: Data curation, Formal analysis, Investigation, Methodology, Writing – original draft, Writing – review & editing. XL: Data curation, Investigation, Methodology, Writing – review & editing. MW: Investigation, Methodology, Writing – review & editing. BX: Data curation, Methodology, Writing – review & editing. YH: Data curation, Writing – review & editing. YL: Conceptualization, Resources, Writing – review & editing. JF: Conceptualization, Funding acquisition, Investigation, Methodology, Project administration, Resources, Supervision, Writing – original draft, Writing – review & editing.
