# Supplementary material for: Nutritional status affects immune function and exacerbates the severity of pulmonary tuberculosis
Source: Front Immunol. 2024 Jul 17;15:1407813. doi: 10.3389/fimmu.2024.1407813 (PMC11288837; doi:10.3389/fimmu.2024.1407813)
Supplement: Supplementary file 1 [file DataSheet_1.docx]

**SUPPLEMENTAL MATERIAL TO**

**Nutritional status affects immune function and exacerbates the severity of pulmonary tuberculosis**

***Abbreviated title:*** Nutritional status, immune function and PTB

Chunli Lu^1,*^, Yunyi Xu^2,*^, Xueya Li^2^, Min Wang^3^, Bei Xie^4^, Yaling Huang^1^, Yan Li^3^, Jiahua Fan^5,#^

^1^Department of Clinical Nutrition, Guangzhou Chest Hospital, Guangdong, 510095, P. R. China

^2^Department of Clinical Laboratory, Guangzhou Chest Hospital, Guangdong, 510095, P. R. China

^3^Department of Tuberculosis, Guangzhou Chest Hospital, Guangdong, 510095, P. R. China

^4^Department of Institute of Tuberculosis, Guangzhou Chest Hospital, Guangdong, 510095, P. R. China

^5^State Key Laboratory of Respiratory Disease, Guangzhou Key Laboratory of Tuberculosis Research, Department of Clinical Nutrition, Guangzhou Chest Hospital, Institute of Tuberculosis, Guangzhou Medical University, Guangdong, 510095, P. R. China

^*^Chunli Lu and Yunyi Xu contributed equally to this work.

Corresponding author:

Dr. Jiahua Fan, [fanjh3@mail2.sysu.edu.cn](mailto:liuyan215@mail.sysu.edu.cn), State Key Laboratory of Respiratory Disease, Guangzhou Key Laboratory of Tuberculosis Research, Department of Clinical Nutrition, Guangzhou Chest Hospital, Institute of Tuberculosis, Guangzhou Medical University

**Table S1. Comparison of the basic characteristics of study participants from discovery and validation cohort**

| **Variables** | **Multiplier effect** | | | |  | **Additive effect** | | |
| --- | --- | --- | --- | --- | --- | --- | --- | --- |
|  | ***β*** | **Wald** | **OR (95% CI)** | P value |  |  | **Estimate** | **95% CI** |
| **Nutritional risk - compromised immune function** | | | | | | | | |
| interaciton | 0.228 | 3.536 | 1.461 (0.985~2.171) | 0.403 |  | RERI | 0.793 | -0.121,1.708 |
| **BMI-PLR** | | | | | | | | |
| interaciton (severity) | 0.00005 | 3.711 | 0.925 (0.853~1.001) | 0.681 |  | RERI (severity) | -0.0002 | -0.0008,0.0002 |
| interaciton (field) | 0.00002 | 8.118 | 0.905 (0.845~0.969) | 0.809 |  | RERI (field) | -0.0001 | -0.0006,0.0004 |
| **PNI-PLR** | | | | | | | | |
| interaciton (severity) | 0.00006 | 15.397 | 0.951 (0.928~0.976) | 0.119 |  | RERI (severity) | 0.0001 | -0.0001,0.0003 |
| interaciton (field) | -0.00001 | 14.042 | 0.977 (0.965~0.989) | 0.663 |  | RERI (field) | -0.0006 | -0.0002,0.0003 |

**Table S2. Association between nutritional or immune status and tuberculosis severity in subjects with complete clinical data**

| **Variables** | | **Unadjusted** | | | |  | **Adjusting for sex, age, diabetes and course of disease** | | | |
| --- | --- | --- | --- | --- | --- | --- | --- | --- | --- | --- |
|  |  | ***β*** | **Wald** | **OR (95% CI)** | ***P*** value |  | ***β*** | **Wald** | **OR (95% CI)** | ***P*** value |
| **Nutritional status** | | | | | | | | | | |
| Tuberculosis severity (field ≥ 3) | BMI | -0.085 | 8.154 | 0.918(0.865~0.973) | 0.004 |  | -0.119 | 13.556 | 0.888 (0.833~0.945) | <0.001 |
|  | NRS | 0.067 | 0.769 | 1.069(0.921~1.242) | 0.380 |  | 0.027 | 0.120 | 1.028 (0.881~1.198) | 0.729 |
|  | PNI | -0.071 | 6.397 | 0.932(0.881~0.983) | 0.011 |  | -0.069 | 5.881 | 0.933 (0.882~0.986) | 0.015 |
|  | ALB | 0.013 | 0.154 | 1.013(0.950~1.082) | 0.695 |  | 0.029 | 0.749 | 1.030 (0.965~1.100) | 0.387 |
|  | PA | 0.000 | 0.064 | 1.000(0.998~1.003) | 0.800 |  | -0.001 | 0.207 | 0.999 (0.997~1.002) | 0.649 |
|  | TRF | 0.001 | 0.463 | 1.001(0.998~1.004) | 0.496 |  | 0.002 | 1.985 | 1.002 (0.999~1.005) | 0.159 |
| Number of pulmonary fields infected with tuberculosis | BMI | -0.083 | 8.102 | 0.920(0.869~0.975) | 0.005 |  | -0.118 | 15.783 | 0.889 (0.838~0.942) | <0.001 |
|  | NRS | 0.096 | 1.618 | 1.101(0.949~1.277) | 0.204 |  | 0.044 | 0.337 | 1.045 (0.902~1.210) | 0.562 |
|  | PNI | -0.014 | 4.143 | 0.986(0.973~0.999) | 0.042 |  | -0.014 | 4.560 | 0.986 (0.973~0.999) | 0.033 |
|  | ALB | -0.051 | 10.12 | 0.950(0.920~0.981) | 0.002 |  | -0.027 | 2.643 | 0.974 (0.943~1.006) | 0.104 |
|  | PA | 0.000 | 0.091 | 1.000(0.998~1.003) | 0.763 |  | -0.001 | 0.430 | 0.999 (0.997~1.002) | 0.512 |
|  | TRF | 0.000 | 0.116 | 1.000(0.997~1.002) | 0.734 |  | 0.001 | 0.491 | 1.001 (0.998~1.004) | 0.484 |
| **Immune status** | | | | | | | | | | |
| Tuberculosis severity (field ≥ 3) | CD4 | 0.001 | 0.385 | 1.001(0.998~NA) | 0.535 |  | 0.000 | 0.040 | 1.000 (0.998~NA) | 0.842 |
|  | CD8 | 0.001 | 0.543 | 1.001(0.999~NA) | 0.461 |  | 0.001 | 0.388 | 1.001 (0.999~NA) | 0.533 |
|  | CD3 | -0.001 | 0.607 | 0.999(NA~1.001) | 0.436 |  | -0.001 | 0.170 | 0.999 (NA~1.002) | 0.681 |
|  | LY | -0.091 | 0.277 | 0.913(0.606~1.044) | 0.599 |  | -0.122 | 0.363 | 0.885 (0.581~1.042) | 0.547 |
|  | NLR | 0.029 | 1.502 | 1.030(0.985~1.082) | 0.220 |  | 0.014 | 0.384 | 1.015 (0.972~1.065) | 0.535 |
|  | PLR | 0.002 | 4.872 | 1.002(1.000~1.003) | 0.027 |  | 0.002 | 5.547 | 1.002 (1.000~1.003) | 0.019 |
| Number of pulmonary fields infected with tuberculosis | CD4 | 0.002 | 1.644 | 1.002(0.999~1.004) | 0.200 |  | 0.001 | 0.318 | 1.001 (0.998~1.003) | 0.573 |
|  | CD8 | 0.001 | 1.465 | 1.001(0.999~1.002) | 0.227 |  | 0.001 | 1.018 | 1.001 (0.999~1.002) | 0.313 |
|  | CD3 | -0.002 | 2.576 | 0.998(0.997~1.000) | 0.109 |  | -0.001 | 0.844 | 0.999 (0.997~1.001) | 0.358 |
|  | LY | -0.040 | 1.282 | 0.961(0.897~1.030) | 0.258 |  | -0.044 | 1.644 | 0.957 (0.895~1.024) | 0.200 |
|  | NLR | 0.035 | 3.299 | 1.035(0.997~1.075) | 0.070 |  | 0.020 | 1.093 | 1.020 (0.983~1.058) | 0.296 |
|  | PLR | 0.001 | 5.01 | 1.001(1.000~1.002) | 0.026 |  | 0.001 | 6.494 | 1.001 (1.000~1.002) | 0.011 |

**Table S3. Characteristics of participants according to pulmonary cavity (n = 952)**

| **Characteristic** | **Total Population** | **Without cavity** | **With cavity** | ***P* value** |
| --- | --- | --- | --- | --- |
|  | **(*n*=952)** | **(*n*=479)** | **(*n*=473)** |  |
| Male n (%) | 645 (67.8%) | 275 (57.4%) | 370 (78.2%) | <0.001 |
| Age (years) | 52.00 [34.00, 64.00] | 48.00 [31.50, 65.00] | 54.00 [40.00, 63.00] | 0.014 |
| Current smoking n (%) | 196 (20.6%) | 83 (17.3%) | 113 ( 23.9%) | 0.013 |
| With diabetes n (%) | 317 (33.3%) | 117 (24.4%) | 200 ( 42.3%) | <0.001 |
| **Nutritional status** | | | | |
| Nutritional risk n (%) | 440 (46.2%) | 193 (40.3%) | 247 (52.2%) | <0.001 |
| NRS score (2002) | 2.00 [1.00, 3.00] | 2.00 [1.00, 3.00] | 3.00 [1.00, 4.00] | <0.001 |
| BMI | 20.15 [18.70, 22.50] | 20.40 [18.80, 22.70] | 19.80 [18.70, 22.20] | 0.011 |
| PNI (g/l) | 43.70 [36.19, 49.30] | 44.90 [37.50, 50.23] | 42.30 [35.00, 47.75] | <0.001 |
| TP (g/l) | 70.23 [64.72, 75.36] | 70.56 [65.01, 75.58] | 69.92 [64.56, 75.18] | 0.565 |
| ALB (g/l) | 36.70 [30.60, 42.00] | 38.00 [32.05, 42.70] | 35.30 [29.40, 40.30] | <0.001 |
| PA (g/l) | 204.61 [150.17, 252.05] | 209.71 [157.77, 254.35] | 197.81 [141.96, 246.37] | 0.014 |
| TRF (g/l) | 210.25 [164.48, 260.80] | 218.22 [169.44, 272.44] | 202.02 [159.92, 248.87] | 0.001 |
| HGB (g/l) | 125.00 [111.00, 138.00] | 126.00 [113.00, 137.50] | 125.00 [109.00, 138.00] | 0.269 |
| **Immune function** | | | | |
| Immunocom promise, n (%) | 526 (55.3%) | 256 (53.4%) | 270 (57.1%) | 0.268 |
| CD4^+^<500, n (%) | 513 (53.9%) | 248 (51.8%) | 265 (56.0%) | 0.194 |
| CD4^+^/CD8^+^<1, n (%) | 86 (9.0%) | 42 (8.8%) | 44 (9.3%) | 0.821 |
| CD4^+^ (cells/ul) | 476.00 [328.00, 664.00] | 484.00 [346.00, 686.00] | 464.00 [308.00, 636.00] | 0.088 |
| CD8^+^ (cells/ul) | 276.00 [181.50, 409.00] | 289.00 [188.00, 433.50] | 268.00 [176.00, 384.00] | 0.043 |
| CD3^+^ (cells/ul) | 796.00 [555.00, 1104.00] | 824.00 [578.00, 1132.00] | 768.00 [536.00, 1072.00] | 0.047 |
| CD4^+^/CD8^+^ | 1.69 [1.30, 2.21] | 1.64 [1.29, 2.19] | 1.72 [1.33, 2.21] | 0.302 |
| LYM (10^9^/L) | 1.24 [0.89, 1.64] | 1.24 [0.90, 1.69] | 1.24 [0.88, 1.61] | 0.514 |
| NLR | 3.78 [2.44, 6.39] | 3.46 [2.32, 5.69] | 4.19 [2.57, 7.10] | <0.001 |
| PLR | 218.56 [151.65, 334.88] | 207.38 [147.51, 303.86] | 230.70 [158.19, 361.84] | 0.021 |
| **Tuberculosis severity** | | | | |
| Smear-positive sputum (%) | 378 (39.7%) | 114 (23.8%) | 264 (55.8%) | <0.001 |
| Bacillary load (%) |  |  |  | <0.001 |
| - | 574 (60.3%) | 365 (76.2%) | 209 (44.2%) |  |
| ± | 63 (6.6%) | 27 (5.6%) | 36 (7.6%) |  |
| 1^+^ | 87 (9.1%) | 28 (5.8%) | 59 (12.5%) |  |
| 2^+^ | 83 (8.7%) | 24 (5.0%) | 59 (12.5%) |  |
| 3^+^ | 75 (7.9%) | 27 (5.6%) | 48 (10.1%) |  |
| 4^+^ | 70 (7.4%) | 8 (1.7%) | 62 (13.1%) |  |
| PTB combined with extrapulmonary tuberculosis (%) | 480 (50.4%) | 251 (52.4%) | 229 (48.4%) | 0.243 |
| Course of disease (month) | 6.00 [2.00, 12.00] | 5.00 [2.00, 12.00] | 9.00 [3.00, 24.00] | <0.001 |
| Drug resistant cases (%) | 397 (41.7%) | 143 (29.9%) | 254 (53.7%) | <0.001 |

**Table S4. Association between nutritional or immune status and tuberculosis severity in subjects with or without cavity**

| **Variables** | | **Unadjusted** | | | |  | **Adjusting for sex, age, smoking, diabetes, bacillary load, course of disease and drug resistant** | | | |
| --- | --- | --- | --- | --- | --- | --- | --- | --- | --- | --- |
|  |  | ***β*** | **Wald** | **OR (95% CI)** | ***P* value** |  | ***β*** | **Wald** | **OR (95% CI)** | ***P* value** |
| **Nutritional status** | | | | | | | | | | |
| With cavity | NRS | 0.083 | 1.79 | 1.087 (0.962~1.229) | 0.181 |  | 0.011 | 0.022 | 1.011 (0.878~1.163) | 0.883 |
|  | BMI | -0.036 | 2.021 | 0.965 (0.918~1.013) | 0.155 |  | -0.100 | 11.122 | 0.905 (0.853~0.959) | 0.001 |
|  | PNI | 0.027 | 1.340 | 1.028 (0.997~1.077) | 0.247 |  | 0.042 | 2.497 | 1.043 (0.997~1.099) | 0.114 |
|  | ALB | -0.062 | 4.808 | 0.940 (0.889~0.982) | 0.028 |  | -0.033 | 1.100 | 0.967 (0.908~1.026) | 0.294 |
|  | PA | 0.001 | 0.849 | 1.001 (0.999~1.003) | 0.357 |  | 0.000 | 0.073 | 1.000 (0.997~1.002) | 0.786 |
|  | TRF | -0.001 | 0.617 | 0.999 (0.997~1.001) | 0.432 |  | 0.000 | 0.051 | 1.000 (0.997~1.002) | 0.821 |
| **Immune status** | | | | | | | | | | |
| With cavity | CD8 | -0.001 | 3.958 | 0.999 (0.998~1.000) | 0.047 |  | -0.002 | 3.652 | 0.998 (0.997~1.000) | 0.049 |
|  | NLR | 0.018 | 1.280 | 1.018 (0.987~1.052) | 0.258 |  | 0.000 | 1.097 | 1.000 (1.000~1.001) | 0.295 |
|  | PLR | 0.000 | 0.367 | 1.000 (0.999~1.001) | 0.544 |  | -0.022 | 1.479 | 0.979 (0.945~1.013) | 0.224 |

**Table S5. Characteristics of participants according to smear sputum (n=952)**

| **Characteristic** | **Total Population** | **Smear-negative sputum** | **Smear-positive sputum** | ***P* value** |
| --- | --- | --- | --- | --- |
|  | **(*n*=952)** | **(*n*=574)** | **(*n*=378)** |  |
| Male n (%) | 645 ( 67.8%) | 360 ( 62.7%) | 285 ( 75.4%) | <0.001 |
| Age (years) | 52.00 [34.00, 64.00] | 51.00 [32.00, 63.00] | 54.00 [40.00, 65.00] | <0.001 |
| Current smoking n (%) | 196 ( 20.6%) | 105 ( 18.3%) | 91 ( 24.1%) | 0.033 |
| With diabetes n (%) | 317 ( 33.3%) | 150 ( 26.1%) | 167 ( 44.2%) | <0.001 |
| **Nutritional status** | | | | |
| Nutritional risk n (%) | 440 ( 46.2%) | 226 ( 39.4%) | 214 ( 56.6%) | <0.001 |
| NRS score (2002) | 2.00 [1.00, 3.00] | 2.00 [1.00, 3.00] | 3.00 [2.00, 4.00] | <0.001 |
| BMI | 20.15 [18.70, 22.50] | 20.20 [18.80, 22.50] | 20.00 [18.70, 22.50] | 0.21 |
| PNI (g/l) | 43.70 [36.19, 49.30] | 46.23 [40.01, 50.77] | 39.12 [32.40, 45.43] | <0.001 |
| TP (g/l) | 70.23 [64.72, 75.36] | 70.18 [64.92, 74.99] | 70.32 [64.59, 75.59] | 0.708 |
| ALB (g/l) | 36.70 [30.60, 42.00] | 38.90 [33.42, 43.20] | 32.80 [27.60, 38.58] | <0.001 |
| PA (g/l) | 204.61 [150.17, 252.05] | 219.93 [167.68, 267.18] | 180.07 [132.88, 226.92] | <0.001 |
| TRF (g/l) | 210.25 [164.48, 260.80] | 220.06 [179.86, 268.67] | 192.50 [141.45, 238.55] | <0.001 |
| HGB (g/l) | 125.00 [111.00, 138.00] | 129.00 [115.00, 140.00] | 121.00 [104.25, 133.75] | <0.001 |
| **Immune function** | | | | |
| Immunocom promise, n (%) | 526 (55.3%) | 291 (50.7%) | 235 (62.2%) | 0.001 |
| CD4^+^<500, n (%) | 513 (53.9%) | 282 (49.1%) | 231 (61.1%) | <0.001 |
| CD4^+^/CD8^+^<1, n (%) | 86 (9.0%) | 49 (8.5%) | 37 (9.8%) | 0.564 |
| CD4^+^ (cells/ul) | 476.00 [328.00, 664.00] | 502.00 [368.00, 696.00] | 414.00 [276.00, 604.00] | <0.001 |
| CD8^+^ (cells/ul) | 276.00 [181.50, 409.00] | 308.50 [208.00, 439.75] | 227.00 [152.00, 351.25] | <0.001 |
| CD3^+^ (cells/ul) | 796.00 [555.00, 1104.00] | 864.00 [621.00, 1164.00] | 700.00 [473.00, 1002.00] | <0.001 |
| CD4^+^/CD8^+^ | 1.69 [1.30, 2.21] | 1.62 [1.26, 2.11] | 1.81 [1.38, 2.37] | <0.001 |
| LYM (10^9^/L) | 1.24 [0.89, 1.64] | 1.32 [0.96, 1.72] | 1.12 [0.77, 1.52] | <0.001 |
| NLR | 3.78 [2.44, 6.39] | 2.98 [2.18, 4.73] | 5.40 [3.45, 9.20] | <0.001 |
| PLR | 218.56 [151.65, 334.88] | 193.31 [135.51, 272.30] | 273.40 [189.52, 435.27] | <0.001 |
| **Tuberculosis severity** | | | | |
| Bacterial load (%) |  |  |  | <0.001 |
| - | 574 (60.3%) | 574 (100.0%) | 0 (0.0%) |  |
| ± | 63 (6.6%) | 0 (0.0%) | 63 (16.7%) |  |
| 1^+^ | 87 (9.1%) | 0 (0.0%) | 87 (23.0%) |  |
| 2^+^ | 83 (8.7%) | 0 (0.0%) | 83 (22.0%) |  |
| 3^+^ | 75 (7.9%) | 0 (0.0%) | 75 (19.8%) |  |
| 4^+^ | 70 (7.4%) | 0 (0.0%) | 70 (18.5%) |  |
| PTB combined with extrapulmonary tuberculosis (%) | 480 (50.4) | 294 (51.2) | 186 (49.2) | 0.552 |
| Course of disease (month) | 6.00 [2.00, 12.00] | 6.00 [2.00, 12.00] | 6.00 [2.00, 24.00] | 0.032 |
| Drug resistant cases (%) | 397 (41.7) | 233 (40.6) | 164 (43.4) | 0.420 |

**Table S6. Association between nutritional or immune status and tuberculosis severity in subjects with or without smear-positive sputum**

| **Variables** | | **Unadjusted** | | | |  | **Adjusting for sex, age, smoke, diabetes and course of disease** | | | |
| --- | --- | --- | --- | --- | --- | --- | --- | --- | --- | --- |
|  |  | ***β*** | **Wald** | **OR (95% CI)** | ***P* value** |  | ***β*** | **Wald** | **OR (95% CI)** | ***P* value** |
| **Nutritional status** | | | | | | | | | | |
| With smear-positive sputum | NRS | 0.067 | 1.237 | 1.069 (0.950~1.204) | 0.266 |  | 0.096 | 2.454 | 1.101 (0.976~1.243) | 0.117 |
|  | PNI | 0.003 | 0.205 | 1.003 (0.989~1.025) | 0.651 |  | 0.001 | 0.024 | 1.001 (0.987~1.022) | 0.876 |
|  | ALB | -0.086 | 28.671 | 0.917 (0.887~0.946) | 0.000 |  | -0.077 | 23.566 | 0.926 (0.895~0.955) | 0.000 |
|  | PA | -0.001 | 0.563 | 0.999 (0.997~1.001) | 0.453 |  | -0.001 | 1.184 | 0.999 (0.996~1.001) | 0.277 |
|  | TRF | 0.000 | 0.005 | 1.000 (0.998~1.003) | 0.942 |  | 0.000 | 0.031 | 1.000 (0.998~1.003) | 0.859 |
| Bacterial load | NRS | 0.088 | 6.904 | 1.092 (1.023~1.166) | 0.009 |  | 0.099 | 8.709 | 1.104 (1.034~1.179) | 0.003 |
|  | PNI | 0.004 | 1.045 | 1.004 (0.996~1.012) | 0.307 |  | 0.003 | 0.692 | 1.003 (0.996~1.011) | 0.406 |
|  | ALB | -0.062 | 52.096 | 0.940 (0.924~0.956) | 0.000 |  | -0.056 | 43.346 | 0.946 (0.930~0.962) | 0.000 |
|  | PA | -0.001 | 3.167 | 0.999 (0.998~1.000) | 0.075 |  | -0.001 | 3.438 | 0.999 (0.997~1.000) | 0.064 |
|  | TRF | 0.001 | 1.280 | 1.001 (0.999~1.002) | 0.258 |  | 0.001 | 0.912 | 1.001 (0.999~1.002) | 0.340 |
| **Immune status** | | | | | | | | | | |
| With smear-positive sputum | CD4 | 0.004 | 9.127 | 1.004 (1.001~1.007) | 0.003 |  | 0.003 | 6.381 | 1.003 (1.001~1.006) | 0.012 |
|  | CD8 | 0.001 | 0.725 | 1.001 (0.999~1.003) | 0.395 |  | 0.000 | 0.379 | 1.000 (0.999~1.003) | 0.538 |
|  | CD3 | -0.003 | 9.274 | 0.997 (0.995~0.999) | 0.002 |  | -0.003 | 7.218 | 0.997 (0.995~0.999) | 0.007 |
|  | LY | 0.441 | 4.962 | 1.554 (1.059~2.291) | 0.026 |  | 0.408 | 4.086 | 1.504 (1.037~2.232) | 0.043 |
|  | NLR | 0.099 | 18.407 | 1.104 (1.057~1.157) | 0.000 |  | 0.083 | 13.567 | 1.087 (1.042~1.138) | 0.000 |
|  | PLR | 0.001 | 3.698 | 1.001 (1.000~1.002) | 0.054 |  | 0.001 | 3.698 | 1.001 (1.000~1.002) | 0.054 |
| Bacterial load | CD4 | 0.002 | 9.162 | 1.002 (1.001~1.003) | 0.003 |  | 0.002 | 6.365 | 1.002 (1.000~1.003) | 0.012 |
|  | CD8 | 0.001 | 5.804 | 1.001 (1.000~1.002) | 0.016 |  | 0.001 | 4.605 | 1.001 (1.000~1.002) | 0.032 |
|  | CD3 | -0.002 | 12.18 | 0.998 (0.997~0.999) | 0.001 |  | -0.002 | 9.764 | 0.998 (0.997~0.999) | 0.002 |
|  | LY | 0.048 | 5.707 | 1.050 (1.009~1.092) | 0.017 |  | 0.042 | 4.383 | 1.043 (1.003~1.085) | 0.037 |
|  | NLR | 0.034 | 12.666 | 1.035 (1.015~1.054) | 0.000 |  | 0.029 | 9.231 | 1.029 (1.010~1.049) | 0.002 |
|  | PLR | 0.001 | 6.897 | 1.001 (1.000~1.001) | 0.009 |  | 0.001 | 7.043 | 1.001 (1.000~1.001) | 0.008 |

**Table S7. Association between nutritional or immune status and tuberculosis severity in subjects stratified according to initial treatment or retreatment**

| **Variables** | | **Unadjusted** | | | |  | **Multivariable adjusting** | | | |
| --- | --- | --- | --- | --- | --- | --- | --- | --- | --- | --- |
|  |  | ***β*** | **Wald** | **OR (95% CI)** | ***P* value** |  | ***β*** | **Wald** | **OR (95% CI)** | ***P* value** |
| **Nutritional status** | | | | | | | | | | |
| **Initial treatment** |  |  |  |  |  |  | **Adjusting for sex, age and diabetes** | | | |
| Tuberculosis severity (field ≥ 3) | NRS | 0.193 | 5.758 | 1.212 (1.037~1.421) | 0.016 |  | 0.156 | 3.561 | 1.169 (0.994~1.375) | 0.059 |
|  | BMI | -0.065 | 3.959 | 0.937 (0.878~0.999) | 0.047 |  | -0.106 | 8.785 | 0.899 (0.837~0.963) | 0.003 |
|  | PNI | -0.090 | 8.518 | 0.914 (0.859~0.970) | 0.004 |  | -0.088 | 7.902 | 0.916 (0.861~0.973) | 0.005 |
|  | ALB | 0.053 | 2.087 | 1.054 (0.982~1.134) | 0.149 |  | 0.066 | 3.166 | 1.068 (0.995~1.151) | 0.075 |
|  | PA | 0.001 | 0.335 | 1.001 (0.998~1.004) | 0.563 |  | 0.000 | 0.048 | 1.000 (0.997~1.003) | 0.827 |
|  | TRF | 0.001 | 0.351 | 1.001 (0.998~1.004) | 0.554 |  | 0.002 | 1.525 | 1.002 (0.999~1.005) | 0.217 |
| Number of pulmonary fields infected with tuberculosis | NRS | 0.243 | 9.675 | 1.276 (1.094~1.487) | 0.002 |  | 0.193 | 6.087 | 1.213 (1.040~1.414) | 0.014 |
|  | BMI | -0.052 | 2.748 | 0.949 (0.893~1.009) | 0.098 |  | -0.096 | 8.830 | 0.908 (0.852~0.968) | 0.003 |
|  | PNI | -0.012 | 3.416 | 0.988 (0.975~1.001) | 0.065 |  | -0.013 | 3.832 | 0.987 (0.975~1.000) | 0.051 |
|  | ALB | -0.038 | 5.243 | 0.963 (0.933~0.995) | 0.022 |  | -0.016 | 0.930 | 0.984 (0.952~1.017) | 0.335 |
|  | PA | 0.001 | 0.947 | 1.001 (0.999~1.004) | 0.331 |  | 0.001 | 0.187 | 1.001 (0.998~1.003) | 0.666 |
|  | TRF | -0.001 | 0.484 | 0.999 (0.996~1.002) | 0.487 |  | 0.000 | 0.05 | 1.000 (0.997~1.003) | 0.830 |
| **Retreatment** |  |  |  |  |  |  | **Adjusting for sex** | | | |
| Tuberculosis severity (field ≥ 3) | NRS | 0.193 | 5.758 | 1.212 (1.037~1.421) | 0.016 |  | 0.191 | 5.593 | 1.210 (1.034~1.419) | 0.018 |
|  | BMI | -0.065 | 3.959 | 0.937 (0.878~0.999) | 0.047 |  | -0.073 | 4.782 | 0.930 (0.871~0.992) | 0.029 |
|  | PNI | -0.090 | 8.518 | 0.914 (0.859~0.970) | 0.004 |  | -0.092 | 8.869 | 0.912 (0.857~0.968) | 0.003 |
|  | ALB | 0.053 | 2.087 | 1.054 (0.982~1.134) | 0.149 |  | 0.060 | 2.617 | 1.062 (0.989~1.143) | 0.106 |
|  | PA | 0.001 | 0.335 | 1.001 (0.998~1.004) | 0.563 |  | 0.000 | 0.081 | 1.000 (0.998~1.003) | 0.775 |
|  | TRF | 0.001 | 0.351 | 1.001 (0.998~1.004) | 0.554 |  | 0.002 | 0.977 | 1.002 (0.998~1.005) | 0.323 |
| Number of pulmonary fields infected with tuberculosis | NRS | 0.243 | 9.675 | 1.276 (1.094~1.487) | 0.002 |  | 0.240 | 9.479 | 1.271 (1.091~1.480) | 0.002 |
|  | BMI | -0.052 | 2.748 | 0.949 (0.893~1.009) | 0.098 |  | -0.062 | 3.904 | 0.940 (0.884~1.000) | 0.049 |
|  | PNI | -0.012 | 3.416 | 0.988 (0.975~1.001) | 0.065 |  | -0.013 | 3.705 | 0.987 (0.975~1.000) | 0.055 |
|  | ALB | -0.038 | 5.243 | 0.963 (0.933~0.995) | 0.022 |  | -0.030 | 3.355 | 0.970 (0.939~1.002) | 0.067 |
|  | PA | 0.001 | 0.947 | 1.001 (0.999~1.004) | 0.331 |  | 0.001 | 0.276 | 1.001 (0.998~1.004) | 0.600 |
|  | TRF | -0.001 | 0.484 | 0.999 (0.996~1.002) | 0.487 |  | 0.000 | 0.01 | 1.000 (0.997~1.003) | 0.944 |
| **Immune status** | | | | | | | | | | |
| **Initial treatment** |  |  |  |  |  |  | **Adjusting for sex, age and diabetes** | | | |
| Tuberculosis severity (field ≥ 3) | CD4 | 0.005 | 1.011 | 1.005 (0.996~1.016) | 0.315 |  | 0.005 | 1.038 | 1.005 (0.996~1.015) | 0.308 |
|  | CD8 | 0.004 | 0.869 | 1.004 (0.996~1.015) | 0.351 |  | 0.005 | 1.117 | 1.005 (0.996~1.015) | 0.291 |
|  | CD3 | -0.005 | 1.115 | 0.995 (0.985~1.004) | 0.291 |  | -0.005 | 1.213 | 0.995 (0.985~1.004) | 0.271 |
|  | LY | -0.046 | 0.289 | 0.955 (0.666~1.044) | 0.591 |  | -0.056 | 0.302 | 0.945 (0.640~1.040) | 0.582 |
|  | NLR | 0.022 | 0.834 | 1.022 (0.978~1.075) | 0.361 |  | 0.012 | 0.243 | 1.012 (0.968~1.064) | 0.622 |
|  | PLR | 0.002 | 6.780 | 1.002 (1.000~1.003) | 0.009 |  | 0.002 | 6.734 | 1.002 (1.000~1.003) | 0.009 |
| Number of pulmonary fields infected with tuberculosis | CD4 | 0.002 | 0.235 | 1.002 (0.993~1.011) | 0.628 |  | 0.002 | 0.192 | 1.002 (0.993~1.011) | 0.661 |
|  | CD8 | 0.001 | 0.075 | 1.001 (0.993~1.010) | 0.784 |  | 0.002 | 0.158 | 1.002 (0.993~1.010) | 0.691 |
|  | CD3 | -0.002 | 0.252 | 0.998 (0.990~1.006) | 0.616 |  | -0.002 | 0.253 | 0.998 (0.990~1.006) | 0.615 |
|  | LY | -0.029 | 0.726 | 0.971 (0.908~1.039) | 0.394 |  | -0.035 | 1.078 | 0.966 (0.904~1.032) | 0.299 |
|  | NLR | 0.032 | 2.674 | 1.032 (0.994~1.072) | 0.102 |  | 0.019 | 1.007 | 1.019 (0.982~1.058) | 0.316 |
|  | PLR | 0.001 | 7.487 | 1.001 (1.000~1.002) | 0.006 |  | 0.001 | 7.530 | 1.001 (1.000~1.002) | 0.006 |
| **Retreatment** |  |  |  |  |  |  | **Adjusting for sex** | | | |
| Tuberculosis severity (field ≥ 3) | CD4 | 0.000 | 0.208 | 1.000 (0.998~1.003) | 0.648 |  | 0.000 | 0.110 | 1.000 (0.998~1.002) | 0.740 |
|  | CD3 | -0.001 | 0.839 | 0.999 (0.998~1.001) | 0.360 |  | -0.001 | 0.588 | 0.999 (0.998~1.001) | 0.443 |
|  | LY | -0.045 | 0.294 | 0.956 (0.669~1.044) | 0.588 |  | -0.053 | 0.300 | 0.949 (0.643~1.041) | 0.584 |
|  | NLR | 0.022 | 0.820 | 1.022 (0.978~1.075) | 0.365 |  | 0.017 | 0.524 | 1.017 (0.973~1.07) | 0.469 |
|  | PLR | 0.002 | 6.686 | 1.002 (1.000~1.003) | 0.010 |  | 0.002 | 6.806 | 1.002 (1.000~1.003) | 0.009 |
| Number of pulmonary fields infected with tuberculosis | CD4 | 0.001 | 0.892 | 1.001 (0.999~1.003) | 0.345 |  | 0.001 | 0.485 | 1.001 (0.999~1.003) | 0.486 |
|  | CD3 | -0.001 | 2.268 | 0.999 (0.998~1.000) | 0.133 |  | -0.001 | 1.507 | 0.999 (0.998~1.000) | 0.220 |
|  | LY | -0.029 | 0.725 | 0.971 (0.908~1.039) | 0.395 |  | -0.034 | 0.999 | 0.967 (0.904~1.033) | 0.318 |
|  | NLR | 0.032 | 2.668 | 1.032 (0.994~1.072) | 0.103 |  | 0.025 | 1.745 | 1.026 (0.988~1.065) | 0.187 |
|  | PLR | 0.001 | 7.472 | 1.001 (1.000~1.002) | 0.006 |  | 0.001 | 7.610 | 1.001 (1.000~1.002) | 0.006 |


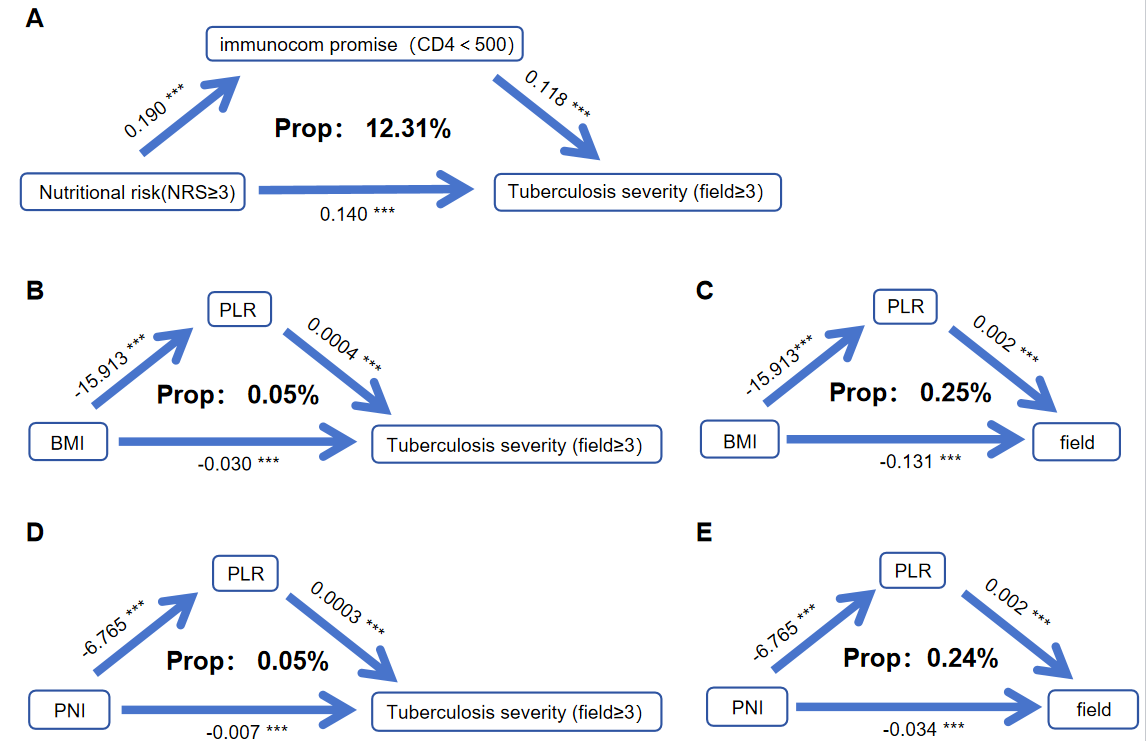


**Figure S1. Nutritional status exacerbates tuberculosis severity by affecting immune function in subjects with complete clinical data.** (A) Mediation linkage among nutritional risk and immunocom promise on tuberculosis. Mediation linkage among BMI and PLR on tuberculosis severity (B) and number of infected lung fields (C), separately. Mediation linkage among PNI and PLR on tuberculosis severity (D) and number of infected lung fields (E), separately. Indirect effects, *P* mediation, and the mediatory effect of each metabolite were denoted. **P*<0.05, ***P*<0.01, and ****P*<0.001.


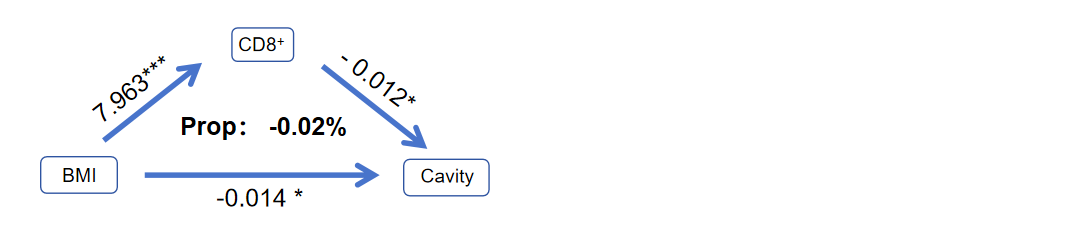


**Figure S2. Nutritional status exacerbates tuberculosis severity by affecting immune function in subjects with or without cavity.** Mediation linkage among BMI and CD8 on tuberculosis severity. Indirect effects, *P* mediation, and the mediatory effect of each metabolite were denoted. **P*<0.05, ***P*<0.01, and ****P*<0.001.


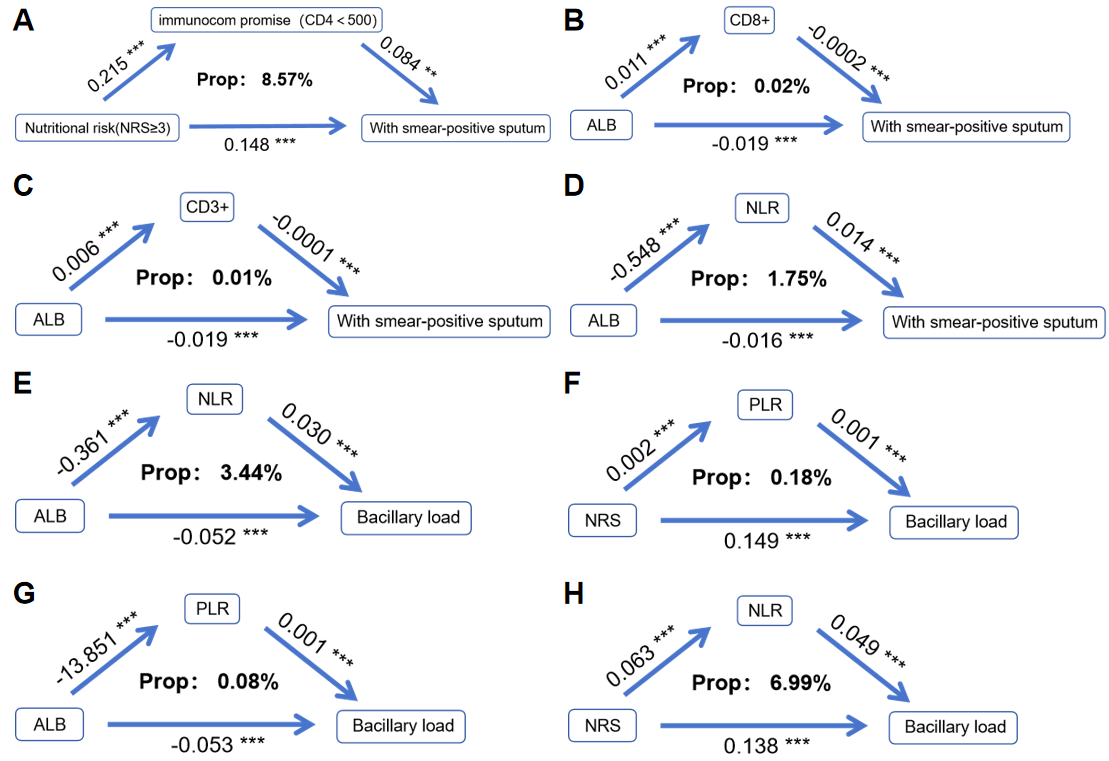


**Figure S3. Nutritional status exacerbates tuberculosis severity by affecting immune function in subjects with or without smear-positive sputum.** (A) Mediation linkage among nutritional risk and immunocom promise on tuberculosis. (B) Mediation linkage among ALB and CD8 on tuberculosis severity. (C) Mediation linkage among ALB and CD3 on tuberculosis severity. Mediation linkage among ALB and NLR on tuberculosis severity (D) and bacillary load (E), separately. (F) Mediation linkage among NRS and PLR on bacillary load. (G) Mediation linkage among ALB and PLR on bacillary load. (H) Mediation linkage among NRS and NLR on bacillary load. Indirect effects, *P* mediation, and the mediatory effect of each metabolite were denoted. **P*<0.05, ***P*<0.01, and ****P*<0.001.


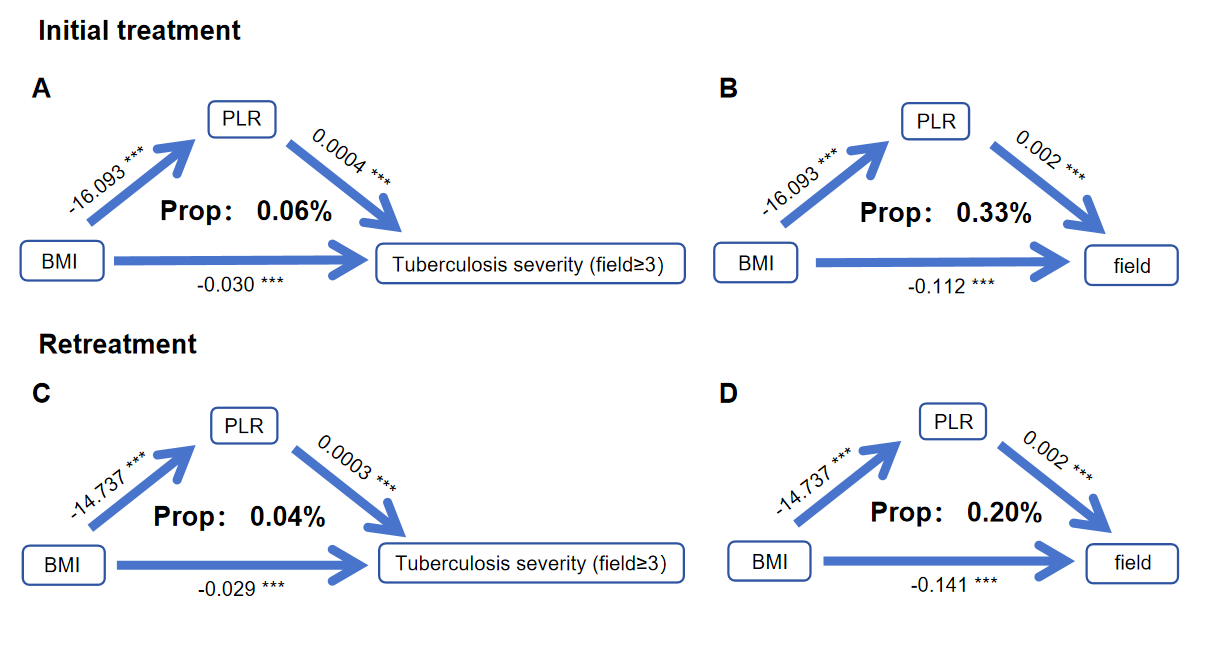


**Figure S4. Nutritional status exacerbates tuberculosis severity by affecting immune function in subjects stratified according to initial treatment or retreatment.** Mediation linkage among BMI and PLR on tuberculosis severity (A) and number of infected lung fields (B) in subjects with initial treatment, separately. Mediation linkage among BMI and PLR on tuberculosis severity (C) and number of infected lung fields (D) in subjects with retreatment, separately. Indirect effects, *P* mediation, and the mediatory effect of each metabolite were denoted. **P*<0.05, ***P*<0.01, and ****P*<0.001.
